# The International Heart Transplant Survival Algorithm (IHTSA): A New Model to Improve Organ Sharing and Survival

**DOI:** 10.1371/journal.pone.0118644

**Published:** 2015-03-11

**Authors:** Johan Nilsson, Mattias Ohlsson, Peter Höglund, Björn Ekmehag, Bansi Koul, Bodil Andersson

**Affiliations:** 1 Department of Clinical Sciences Lund, Cardiothoracic Surgery, Lund University and Skåne University Hospital, Lund, Sweden; 2 Department of Astronomy and Theoretical Physics, Computational Biology and Biological Physics, Lund University, Lund, Sweden; 3 Competence Center for Clinical Research, Lund University and Skåne University Hospital, Lund, Sweden; 4 Department of Clinical Sciences Lund, Cardiology, Lund University and Skåne University Hospital, Lund, Sweden; 5 Department of Clinical Sciences Lund, Surgery, Lund University and Skåne University Hospital, Lund, Sweden; University of Toledo, UNITED STATES

## Abstract

**Background:**

Heart transplantation is life saving for patients with end-stage heart disease. However, a number of factors influence how well recipients and donor organs tolerate this procedure. The main objective of this study was to develop and validate a flexible risk model for prediction of survival after heart transplantation using the largest transplant registry in the world.

**Methods and Findings:**

We developed a flexible, non-linear artificial neural networks model (IHTSA) and classification and regression tree to comprehensively evaluate the impact of recipient-donor variables on survival over time. We analyzed 56,625 heart-transplanted adult patients, corresponding to 294,719 patient-years. We compared the discrimination power with three existing scoring models, donor risk index (DRI), risk-stratification score (RSS) and index for mortality prediction after cardiac transplantation (IMPACT). The accuracy of the model was excellent (C-index 0.600 [95% CI: 0.595–0.604]) with predicted versus actual 1-year, 5-year and 10-year survival rates of 83.7% versus 82.6%, 71.4% – 70.8%, and 54.8% – 54.3% in the derivation cohort; 83.7% versus 82.8%, 71.5% – 71.1%, and 54.9% – 53.8% in the internal validation cohort; and 84.5% versus 84.4%, 72.9% – 75.6%, and 57.5% – 57.5% in the external validation cohort. The IHTSA model showed superior or similar discrimination in all of the cohorts. The receiver operating characteristic area under the curve to predict one-year mortality was for the IHTSA: 0.650 (95% CI: 0.640–0.655), DRI 0.56 (95% CI: 0.56–0.57), RSS 0.61 (95% CI: 0.60–0.61), and IMPACT 0.61 (0.61–0.62), respectively. The decision-tree showed that recipients matched to a donor younger than 38 years had additional expected median survival time of 2.8 years. Furthermore, the number of suitable donors could be increased by up to 22%.

**Conclusions:**

We show that the IHTSA model can be used to predict both short-term and long-term mortality with high accuracy globally. The model also estimates the expected benefit to the individual patient.

## Introduction

Heart transplantation is life-saving for patients with end-stage heart disease. A limiting factor is the lack of donor organs. Unfortunately, currently we do not make use of more than one-third of the organs offered, partly due to donors being unsuitable and partly due to uncertainty in the risk of early and late graft dysfunction [1]. The acceptance of the heart is usually done before a detailed organ inspection has been performed. With an improved prediction of outcome based on the characteristics of both the recipient and donor, we can have more confidence in the post-transplantation performance and it may be possible to improve survival and increase the number of organs that can be used [2].

Basic immunological incompatibility between humans is a well-known risk factor for premature death after organ transplantation [3]. Today, ABO blood group compatibility, recipient and donor size, gender, and urgency are the main criteria for matching of potential recipients with an appropriate donor [4]. Retrospective studies have indicated that better matching results in fewer episodes of immunological rejection and infection, with an overall improvement in survival rate [5]. In an effort to improve the matching between donor and recipient, the effect on the outcome of different risk factors has been studied and risk-stratification models designed to predict post-transplantation performance have been published [6–10]. However, these models are limited to predicting short-term mortality and are derived from a regional patient cohort. External validation is often lacking. The multivariable analysis used in these studies assumes linear relationships with the risk of an event to be occurring. More complex analyses, e.g. involving interaction between several of the variables or complex non-linear relationships, are lacking. To date, no studies have derived a global heart transplantation survival prediction model or comprehensively evaluated the relative importance of existing matching criteria for long-term survival or constructed a model that can quantify post-transplantation performance in term of years gained, for this patient group.

The main objective of this study was to develop and validate a flexible risk model for prediction of survival after heart transplantation using an international heart transplant database, The International Society for Heart and Lung Transplantation (ISHLT) registry [11]. Further objectives were to make the model easily interpretable and to visualize the relative importance and interactions between the recipient-donor match variables by constructing a decision tree.

## Materials and Methods

### Data source

The ISHLT registry is the largest repository of heart transplant data in the world [11]. The registry includes data stored since 1980’s and contains almost 400 variables, including pre-transplantation, transplantation, discharge, and follow-up variables. The data is collected through three mechanisms: manual data entry via a web-based data entry system, electronic download of data from individual centers, or via a data sharing arrangement with a regional/national Organ Procurement Organization or Organ Exchange Organization. At present nine data collectives, such as United Network for Organ Sharing, Eurotransplant, and Scandia Transplant, have agreements with the ISHLT registry and provide data from 345 participating institutions. The total number of registered heart transplants was 111,068, June 30, 2012. The quality of the registry data is dependent on the accuracy and completeness of the centres reporting and collecting the data. Various quality control measures are used by the registry to avoid including low-quality or incomplete data in the main dataset. Post-transplant information is reported annually until the time of death. The date of death after heart transplantation is provided by each reporting transplant center. A complete list of data collectives, participating institutions and ISHLT registration data elements are available at http://www.ishlt.org/registries/.

The Nordic Thoracic Transplantation Database (NTTD) is a registry within the Scandia Transplant organization, incorporating all thoracic transplantations performed in the five Nordic countries (http://www.scandiatransplant.org). The registry has been in existence since 1983 and consists of more than 400 variables, and it is mandatory for all centers performing transplantations [12]. The total number of registered heart transplants was 2,853, December 31, 2013.

### Study population

Data on heart donors and the corresponding recipient who were transplanted between January 1, 1994 and December 31, 2010 were collected from the ISHLT registry (n = 68,085). Pediatric cases (recipients younger than 18 years, n = 9,120) and those with incomplete mandatory data (diagnosis, blood group, age, gender, duration of follow-up, and/or cause of death, n = 2,340) were excluded. The final study population comprised 56,625 transplants (55,956 patients). The latest annual follow-up was on October 9, 2011. From the NTTD registry, data on heart donors and the corresponding recipients who were transplanted between January 1, 1996 and December 31, 2010 were collected (n = 1,635). Pediatric cases (n = 189) and those with incomplete mandatory data (n = 161) were excluded. The final study population comprised 1,285 transplants (1,271 patients). The latest annual follow-up was on September 30, 2011.

### Study design

The variables used in the model were based on information available at the time of transplantation (N = 62). Variables including more than two categories were dichotomized to n category-1 variables ending up in 77 variables used. The primary endpoint was all cause cumulative mortality during the study period. The patients whose data were obtained from the ISHLT registry, who were transplanted between January 1, 1994 and December 31, 2008, were randomly divided into a derivation cohort (DC) (n = 41,780) and an independent internal validation cohort (IVC) (n = 8,569). Patients transplanted during a later time era (between January 1, 2009 and December 31, 2010) were used as a temporal validation cohort (TVC) (n = 6,276). As a regional validation cohort, data collected from the NTTD (n = 1,285) were used. The DC was used to develop the survival model, called the International Heart Transplantation Survival Algorithm (IHTSA), and to quantify the relative importance of various recipient-donor combinations and their expected survival.

### Statistical analysis

Statistical analyses were performed using the Stata MP statistical package version 13 (2013) (StataCorp LP, College Station, TX). Data are presented as means with standard deviation, median with interquartile range (IQR) and frequency as appropriate. Unpaired Mann-Whitney U-tests were used to compare continuous variables and χ^2^ tests were used to compare categorical variables among groups. The discrimination for long-term survival was evaluated using Harrell’s concordance index (C-index) [13]. To compare different C-indexes, the Somers’ D statistic was used [14]. Log-rank test was used to compare Kaplan-Meier survival curves. Logistic regression was performed to calibrate the models on one-year mortality. Using the Hosmer-Lemeshow goodness-of-fit test, we assessed predictive accuracy. The discriminatory power for one-year mortality was assessed by calculating the area under the receiver-operating curve (AUROC). To compare different areas, non-parametric approach described by DeLong was used [15]. The Donor Risk Index (DRI), Index for Mortality Prediction After Cardiac Transplantation (IMPACT), and Risk Stratification Score (RSS) were pre-calculated for each patient using the previously published algorithms [6,7,9].

### Imputation of missing data

As in all multi-institutional registries the ISHLT registry contains missing data. We evaluated three different imputation techniques; multiple imputation using nearest neighbor, multiple imputation using probability imputation with and without stratification by time era, and case-wise deletion of patients with more than 30% missing values together with multiple imputation using probability imputation. The case-wise deletion method resulted in the best performance for the derivation cohort but weak generalization in the validation cohorts. The probability imputation technique stratified by time era resulted in the best overall performance. Each missing data was imputed 10 times with a random existing data point from another patient, which resulted in 10 study cohorts with a variation in variables that had missing data. The survival model was trained and validated using these 10 cohorts, which resulted in a counterbalance of random fluctuations and utilized the ensemble approach.

### Survival model

**Fig 1:**
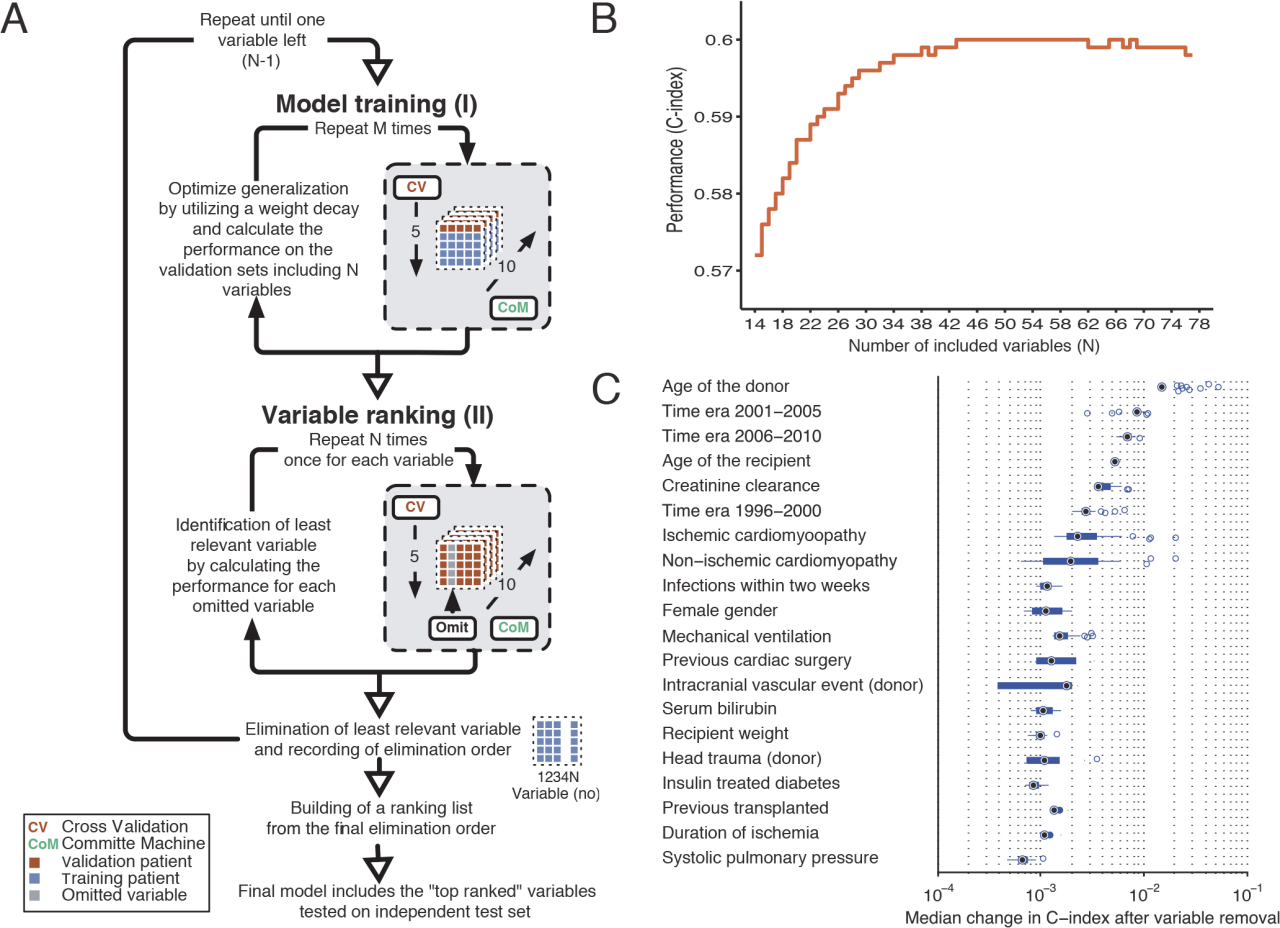
Schematic illustration of the variable-ranking process for the derivation cohort. Panel (A). Step I: training of the survival model using fivefold cross-validation and a committee machine with 10 members. Step II: variable-ranking using the trained survival model from step I. Each variable is omitted from the model and the decrease in performance is recorded. The variable resulting in the least reduction in performance is removed. Steps I–II are repeated until only one variable is left. A ranking list is constructed using the elimination order. Panel (B). Performance as a function of number of variables included. The C-index is plotted against the number of input variables, where order is in terms of decreasing importance. Variables with a high index number are least important. Panel (C). The relative importance of 20 of the 43 top-ranked variables. The box plot for each variable is created from the series of C-index changes that was the result of removal of the variable during the ranking procedure.

The survival model used was a flexible non-linear generalization of the standard Cox proportional-hazard model. It follows the principles described by Biganzoli, with the extension of using ensembles of artificial neural networks (ANNs) instead of a single prediction model [16]. Unadjusted predictors of cumulative mortality were identified by performing a ranking of the risk variables. Variables involving both the donor and recipient (i.e. blood group, body size, gender, and age) were predefined as mandatory variables (N = 13) and were not included in the ranking procedure. Fig. 1A shows a schematic illustration of the ANN calibration and the variable-ranking process for the DC. Fig. 1B illustrates the performance in relation to the number of risk variables included in the model. The final model included 43 inputs, 18 hidden nodes, 25 time intervals and 10 committee members in the ensemble. The time-dependent hazard ratios for the risk variables were defined in a similar way as described by others [17,18]. By changing the risk variable from absent to present and calculating the hazard for the two conditions at each time interval, a time dependent hazard ratio for the specific risk variable of each patient could be determined. For continuous variables the increment was ½ IQR. The hazard ratio for the specific variable was then obtained by computing the geometric mean of the hazard ratio from all patients. The 95% confidence intervals hazard ratio was calculated using the bootstrap technique (N = 10,000). Development of the international heart transplantation survival algorithm (IHTSA) model and simulation (e.g. the sub-group analysis with matched cohorts) was performed using a high-performance computer cluster with MATLAB Distribution Computing Server 2010a, Neural Network Toolbox and Statistical Toolbox (MathWorks, Natick, MA). Details of the ANN models can be found in the S1 Methods.

### Calibration of the survival model

Calibration of each individual ANN was accomplished by minimizing a cross-entropy error function using resilient back-propagation. To find the optimal regularization parameter and the optimal number of hidden nodes for the ANN 5-fold cross-validation was utilized. The number of hidden nodes was determined based on experiments starting with a single node and increasing the number of nodes until the highest accuracy was found for the validation sets. By a similar procedure, a weight decay term was chosen to optimize the validation performance. When all parameters were set a new calibration using the full training dataset was performed. Throughout the model calibration the ensemble approach was used utilizing 10 different datasets, obtained from the missing data imputation technique. The derivation cohort was used to calibrate and identify the optimal architecture for the ANN. Details of the calibration can be found in the S1 Methods.

### Decision tree

The relative importance of the recipient-donor combinations and their expected survival were obtained using a classification and regression tree (CART) model that was fitted to the predicted median survival time [19]. The survival time was pre-calculated and adjusted to the later time era for each patient using the IHTSA model, before the decision tree was constructed. The importance of individual explanatory variables was determined by measuring the proportion of variability accounted for by splits associated with each explanatory variable. Pruning optimized the size of the tree.

### Organ allocation simulation

In order to evaluate the clinical consequence of a new organ allocation policy, we designed a simulation model. The model records the number of possible transplantations and the predicted survival when the IHTSA model or a criterion based clinical model were used [4,20]. For the IHTSA model the recipient with the best-predicted survival was chosen. For the clinical model all recipient-donors who fulfilled the two clinical matching criteria compatible blood-group match and a recipient/donor weight ±20%, were defined as possible recipients. Among those the recipients were prioritized in the following order: 1) identical blood group and both recipient and donor age ≤35 years, 2) identical blood group and a donor age < recipient age +15 years. If PVR was >3.0, the donor must have a 0–15% larger weight compared to the recipient and no donation from female to male was allowed.

The simulation was started by creating a waiting list, including the predefined number (NW = 10, 20, 30, 40, 50, 100 or 200) of randomly selected recipients from one of the validation cohorts. Next a donor was randomly selected from the same validation cohort. The blood-group compatibility was tested for all patients on the previously created waiting list. A donor with at least one successful recipient blood-group match was randomized to be allocated using one of the allocation model (IHTSA respectively clinical model) or the control model (random selection of blood-group compatibility recipients). If two or more recipients reached the same priority a random selection was performed. If a recipient-donor match took place the estimated median survival time for the selected recipient-donor pair was calculated and the data recorded. Then the waiting list was updated with a new recipient, and the procedure was repeated by randomly selecting a new donor.

### Web-based calculator

The developed IHTSA model was deployed as a small web application (http://www.ihtsa.med.lu.se) where users can enter clinical data and get response in terms of estimated survival curves together with relevant survival and mortality numbers. The application was implemented as a simple CGI application using Perl (version 5.16.0) and Matlab (version 2010A) programs for running the survival models. Using a modular approach new survival models can easily be implemented and tested in the web application.

### Ethics Statement

The ISHLT Registry approved the protocol and provided data. The Ethics Committee for Clinical Research at Lund University, Sweden approved the study protocol (2011/126). The data was anonymized and de-identified prior to analysis and the institutional review board waived the need for written informed consent from the participants.

## Results

### Characteristics of the derivation and validation cohorts

The derivation and validation cohorts comprised 294,719 patient-years (median survival time 11.1 years, IQR 3.6–17.4), with a mean duration of follow-up of 5.2 ± 4.6 years (median 4.1, range 0–17.8 years). The one-year mortality was 18% (n = 9,380). A total of 21,502 patients (38%) died during follow-up. The DC and IVC were comparable with regard to baseline demographic data, preoperative hemodynamic status, laboratory values, and immunology (see Tables 1 and 2; complete listing available in S1 Table). The Kaplan-Meier survival estimate were similar (p = 0.823), with a median survival time of 11.1 and 10.9 years, respectively. The TVC and the external NTTD were different in several of the baseline demographic data. As shown in Tables 1 and 2, the patients had more co-morbidity in the TVC, and the donors were significantly older in the NTTD than in the DC.

**Table 1:** Baseline characteristics of recipients in the derivation and validation cohorts.

	ISHLT					NTTD	
	DC	IVC		TVC			
	n = 41,780	n = 8,569	p Value	n = 6,276	p Value	n = 1,285	p Value
Demographic data							
Age (years)	45 **54** 60	45 **54** 60	0.430	45 **54** 61	0.406	43 **52** 58	0.001
Female gender	8,463 (20%)	1,827 (21%)	0.026	1,485 (24%)	0.001	286 (22%)	0.079
Height (cm)	168 **174** 180	168 **175** 180	0.350	168 **175** 180	0.018	170 **176** 182	0.001
Weight (kg)	66 **77** 88	67 **77** 88	0.470	69 **80** 91	0.001	66 **77** 86	0.085
Ischemic cardiomyopathy	17,670 (42%)	3,630 (42%)	0.990	2,166 (35%)	0.001	450 (36%)	0.001
Non-ischemic cardiomyopathy	19,265 (46%)	3,946 (46%)	0.918	3,213 (51%)	0.001	613 (50%)	0.005
Congenital	867 (2%)	180 (2%)	0.881	203 (3%)	0.001	49 (4%)	0.001
Graft failure	846 (2%)	168 (2%)	0.699	151 (2%)	0.001	5 (0%)	0.001
Diabetes mellitus^#^	4,802 (20%)	1,006 (21%)	0.750	1,059 (25%)	0.001	82 (11%)	0.001
Hypertension^†^	9,185 (38%)	1,904 (38%)	0.990	1,526 (44%)	0.001	141 (19%)	0.001
Infection within two weeks^‡^	2,326 (10%)	493 (10%)	0.600	410 (10%)	0.670	40 (5%)	0.001
Antiarrhythmic drugs prior to transplant	8,472 (37%)	1,755 (37%)	0.860	1,255 (39%)	0.007	263 (36%)	0.800
Amiodarone prior to transplant	5,787 (26%)	1,223 (26%)	0.410	993 (32%)	0.001	172 (23%)	0.190
Previous blood transfusion	7,574 (51%)	1,569 (51%)	0.360	966 (86%)	0.001		
Previously transplanted*	1,160 (3%)	224 (3%)	0.910	200 (3%)	0.300	5 (0%)	0.001
Previous cardiac surgery	6,594 (28%)	1,371 (28%)	0.410	2,465 (60%)	0.001	305 (24%)	0.001
ICU	9,679 (38%)	1,989 (38%)	0.470	1,043 (26%)	0.001	97 (12%)	0.001
Mechanical ventilation	731 (3%)	151 (3%)	0.980	138 (3%)	0.430		
ECMO	106 (0%)	23 (0%)	0.840	39 (1%)	0.001		
Ventricular assist device	4,367 (26%)	902 (26%)	0.830	1,429 (34%)	0.001		
Transplant era			0.200				0.001
1991–1995	6,029 (14%)	1,259 (15%)					
1996–2000	14,804 (35%)	2,949 (34%)				450 (35%)	
2001–2005	13,078 (31%)	2,682 (31%)				451 (35%)	
2006–2010	7,869 (19%)	1,679 (20%)		6,276 (100%)		384 (30%)	
Hemodynamic status							
PVR (wood units)	1.4 **2.1** 3.2	1.5 **2.2** 3.2	0.140	1.4 **2.2** 3.1	0.620	1.8 **2.6** 3.7	0.001
SPP (mmHg)	33 **42** 54	33 **42** 54	0.930	33 **41** 52	0.001		
Laboratory values							
Creatinine (μmol/l)	88 **106** 132	88 **106** 132	0.910	85 **106** 132	0.001	88 **103** 125	0.001
Serum bilirubin (mg/dl)	0.60 **0.80** 1.30	0.60 **0.80** 1.30	0.470	0.50 **0.80** 1.30	0.003	0.71 **1.12** 1.71	0.001
Immunology status							
PRA > 10%	1,841 (8%)	370 (8%)	0.590	402 (11%)	0.001	87 (7%)	0.047
HLA-DR			0.520		0.086		0.004
0 mismatch	720 (4%)	163 (4%)		142 (5%)		19 (3%)	
1 mismatch	7,324 (41%)	1,496 (40%)		1,169 (40%)		339 (46%)	
2 mismatches	9,934 (55%)	2,060 (55%)		1,646 (56%)		373 (51%)	
Recipient blood group			0.440		0.001		0.019
A	18,304 (44%)	3,752 (44%)		2,629 (42%)		618 (48%)	
AB	2,737 (7%)	539 (6%)		500 (8%)		72 (6%)	
B	5,300 (13%)	1,051 (12%)		859 (14%)		158 (12%)	
O	15,439 (37%)	3,227 (38%)		2,288 (36%)		437 (34%)	

*a **b** c* represent the lower quartile *a*, the median ***b***, and the upper quartile *c* for continuous variables. Numbers within parenthesis are frequencies. The numbers were calculated from patients with data available.

^#^Drug or insulin treated diabetes mellitus.

^†^Drug treated systemic hypertension.

^‡^Infection requiring intravenous antibiotic therapy within two weeks prior to transplant.

*Previous transplant—previous kidney, liver, pancreas, pancreas islet cells, heart, lung, intestine and/or bone marrow transplant. COPD, chronic obstructive pulmonary disease; DC, derivation cohort; HLA, human leukocyte antigen; ISHLT, international society for heart and lung transplantation; IVC, internal validation cohort; NTTD, Nordic thoracic transplantation database; PRA, panel reactive antibody; PVR, pulmonary vascular resistance; SPP, systolic pulmonary pressure. TVC, temporal validation cohort. Statistical tests: Unpaired Mann-Whitney U-tests or and χ^2^ tests.

**Table 2:** Baseline characteristics of donors in the derivation and validation cohorts.

	ISHLT					NTTD	
	DC	IVC		TVC			
	n = 41,780	n = 8,569	p Value	n = 6,276	p Value	n = 1,285	p Value
Demographic data							
Age (years)	22 **34** 44	22 **33** 44	0.094	22 **34** 46	0.001	29 **41** 50	0.001
Female gender	13,011 (31%)	2,657 (31%)	0.810	1,981 (32%)	0.500	451 (35%)	0.003
Weight (kg)	67 **75** 76	67 **75** 76	0.710	70 **80** 90	0.001	70 **75** 85	0.370
Ischemic time (min)	140 **183** 225	141 **183** 225	0.180	152 **195** 235	0.001	120 **185** 219	0.001
CODD: Head trauma	17,497 (51%)	3,673 (53%)	0.010	2,233 (44%)	0.001	285 (22%)	0.001
CODD: Cerebrovascular event	10,482 (31%)	2,2064 (30%)	0.058	1,400 (26%)	0.001	733 (57%)	0.001
Donor blood group			0.220		0.001		0.014
A	16,802 (40%)	3,446 (40%)		2,315 (37%)		544 (42%)	
AB	1,266 (3%)	257 (3%)		312 (5%)		20 (2%)	
B	4,226 (10%)	806 (9%)		675 (11%)		132 (10%)	
O	19,486 (47%)	4,060 (47%)		2,974 (47%)		589 (46%)	

*a **b** c* represent the lower quartile *a*, the median ***b***, and the upper quartile *c* for continuous variables. Numbers within parenthesis are frequencies. N is the number of non––missing values. The numbers were calculated from patients with data available. CODD, cause of donor death; DC, derivation cohort; ISHLT, international society for heart and lung transplantation; IVC, internal validation cohort; NTTD = Nordic thoracic transplantation database; PRA, panel-reactive antibody; PVR, pulmonary vascular resistance; SPP, systolic pulmonary pressure. TVC, temporal validation cohort. Statistical tests: Unpaired Mann-Whitney U-tests or and χ^2^ tests.

### Derivation of the survival model

The final IHTSA model included 43 variables (Tables 3 and 4), resulting in a Harrell’s C-index of 0.600 (95% CI: 0.595–0.604) in the DC. The AUROC for prediction of one-year mortality was 0.650 (95% CI: 0.640–0.655). Of the 56,625 patients in total 56,311 received a unique survival time prediction with the IHTSA model. As illustrated in Fig. 1C, the age of the donors and recipients, recipient creatinine levels, diagnosis, time era, infections within two weeks before transplantation, gender, and use of mechanical ventilation prior to transplantation influenced the discrimination of the model the most.

**Table 3:** Time-dependent hazard ratios for the 32 recipient risk variables included in the IHTSA model.

	Hazard ratio at 1 year		Hazard ratio at 5 years		Hazard ratio at 10 years	
Demographic data						
Age (per 8 years)	1.06	(1.055–1.056)	1.11	(1.113–1.115)	1.20	(1.199–1.202)
Female gender	1.11	(1.106–1.108)	1.11	(1.109–1.112)	1.08	(1.073–1.077)
Height (per 6 cm)	0.99	(0.986–0.987)	0.99	(0.991–0.992)	1.00	(0.998–0.998)
Weight (per 10 kg)	1.02	(1.018–1.018)	1.02	(1.024–1.024)	1.03	(1.025–1.025)
Ischemic cardiomyopathy	1.03	(1.025–1.027)	1.14	(1.137–1.139)	1.29	(1.292–1.295)
Non-ischemic cardiomyopathy	0.94	(0.943–0.944)	0.99	(0.992–0.994)	1.08	(1.078–1.080)
Congenital	1.05	(1.045–1.048)	0.96	(0.960–0.964)	0.78	(0.777–0.782)
Graft failure	1.16	(1.159–1.162)	1.36	(1.356–1.361)	1.49	(1.490–1.497)
Diabetes mellitus^#^	1.09	(1.090–1.092)	1.15	(1.147–1.149)	1.19	(1.186–1.188)
Hypertension^†^	1.07	(1.068–1.069)	1.07	(1.066–1.067)	1.05	(1.054–1.055)
Infection within two weeks^‡^	1.15	(1.145–1.146)	1.15	(1.148–1.150)	1.12	(1.115–1.118)
Antiarrhythmic drugs prior to transplant	0.99	(0.991–0.992)	0.99	(0.984–0.985)	1.01	(1.005–1.006)
Amiodarone prior to transplant	0.97	(0.970–0.971)	0.95	(0.947–0.949)	0.92	(0.922–0.924)
Previous blood transfusion	1.03	(1.028–1.029)	0.99	(0.993–0.995)	0.99	(0.984–0.985)
Previously transplanted*	1.59	(1.585–1.589)	1.41	(1.411–1.416)	1.13	(1.127–1.133)
Previous cardiac surgery	1.07	(1.067–1.069)	1.01	(1.009–1.011)	0.97	(0.968–0.970)
Intensive care unit	1.06	(1.062–1.063)	1.05	(1.044–1.046)	1.03	(1.031–1.033)
Mechanical ventilation	1.24	(1.237–1.239)	1.07	(1.065–1.068)	0.92	(0.920–0.923)
ECMO	1.34	(1.338–1.343)	1.28	(1.274–1.280)	0.79	(0.785–0.792)
Ventricular assist device	1.03	(1.031–1.032)	0.99	(0.991–0.992)	0.95	(0.944–0.945)
Transplant era						
1996–2000	0.84	(0.839–0.840)	0.83	(0.827–0.828)	0.84	(0.834–0.836)
2001–2005	0.74	(0.736–0.737)	0.73	(0.725–0.726)	0.70	(0.700–0.702)
2006–2010	0.73	(0.731–0.732)	0.74	(0.740–0.742)	0.71	(0.704–0.707)
Hemodynamic status						
SPP (per 10 mmHg)	1.02	(1.019–1.019)	1.01	(1.006–1.006)	1.00	(0.997–0.997)
PVR (per wood units)	1.01	(1.009–1.009)	1.01	(1.005–1.005)	1.00	(1.001–1.001)
Laboratory values						
Creatinine (per 22 μmol/l)	1.03	(1.031–1.031)	1.02	(1.021–1.021)	1.01	(1.010–1.010)
Serum bilirubin (per 0.4 mg/dl)	1.01	(1.006–1.006)	1.00	(1.003–1.003)	1.00	(1.000–1.000)
Immunology status						
PRA > 10%	1.09	(1.086–1.087)	1.10	(1.103–1.105)	1.07	(1.065–1.067)
HLA–DR, 2 mismatch	1.06	(1.063–1.064)	1.05	(1.050–1.051)	1.05	(1.045–1.046)
Recipient blood group						
A	0.95	(0.949–0.951)	0.96	(0.955–0.957)	1.02	(1.020–1.023)
B	1.03	(1.031–1.033)	1.02	(1.020–1.021)	1.01	(1.010–1.013)
O	1.04	(1.035–1.037)	1.02	(1.022–1.024)	1.07	(1.065–1.068)

The data are median hazard ratio together with 95% confidence interval for the different time points estimated from 10,000 bootstrap samples.

^#^Drug or insulin treated diabetes mellitus.

^†^Drug treated systemic hypertension.

^‡^Infection requiring intravenous antibiotic therapy within two weeks prior to transplant.

*Previous transplant—previous kidney, liver, pancreas, pancreas islet cells, heart, lung, intestine and/or bone marrow transplant. ECMO, extracorporeal membrane oxygenation; ICU, intensive care unit; ITHSA, international heart transplantation survival algorithm; HLA, human leukocyte antigen; PRA, panel reactive antibody; PVR, pulmonary vascular resistance; SPP, systolic pulmonary pressure.

**Table 4:** Time-dependent hazard ratios for the 11 donor risk variables included in the IHTSA model.

	Hazard ratio at 1 year		Hazard ratio at 5 years		Hazard ratio at 10 years	
Demographic data						
Age (per 11 years)	1.17	(1.172–1.173)	1.11	(1.111–1.112)	1.08	(1.075–1.075)
Female gender	1.02	(1.019–1.020)	1.04	(1.038–1.039)	1.04	(1.040–1.042)
Weight (per 10 kg)	0.99	(0.994–0.994)	1.02	(1.018–1.018)	1.03	(1.030–1.030)
Duration of ischemia (per 42 min)	1.03	(1.025–1.026)	1.01	(1.008–1.008)	1.00	(0.997–0.998)
CODD: Head trauma	0.90	(0.896–0.898)	1.00	(0.998–1.000)	1.09	(1.085–1.087)
CODD: Cerebrovascular event	0.89	(0.890–0.891)	0.97	(0.969–0.971)	1.05	(1.050–1.052)
Donor blood group						
B	1.05	(1.044–1.046)	1.07	(1.070–1.073)	1.09	(1.092–1.096)
AB	1.13	(1.126–1.129)	1.16	(1.154–1.157)	1.14	(1.135–1.140)
O	0.96	(0.960–0.961)	0.98	(0.983–0.985)	0.99	(0.991–0.993)
Recipient-donor weight ratio (per 0.14 units)	1.02	(1.015–1.015)	1.01	(1.014–1.014)	1.02	(1.016–1.016)
Recipient-donor height ratio (per 0.04 units)	0.99	(0.991–0.991)	1.00	(0.996–0.996)	1.00	(0.999–0.999)

The data are hazard ratio together with 95% confidence interval for the different time points estimated from 10,000 bootstrap samples. CODD, cause of donor death; ITHSA, international heart transplantation survival algorithm.

### Validation of the IHTSA model

**Fig 2:**
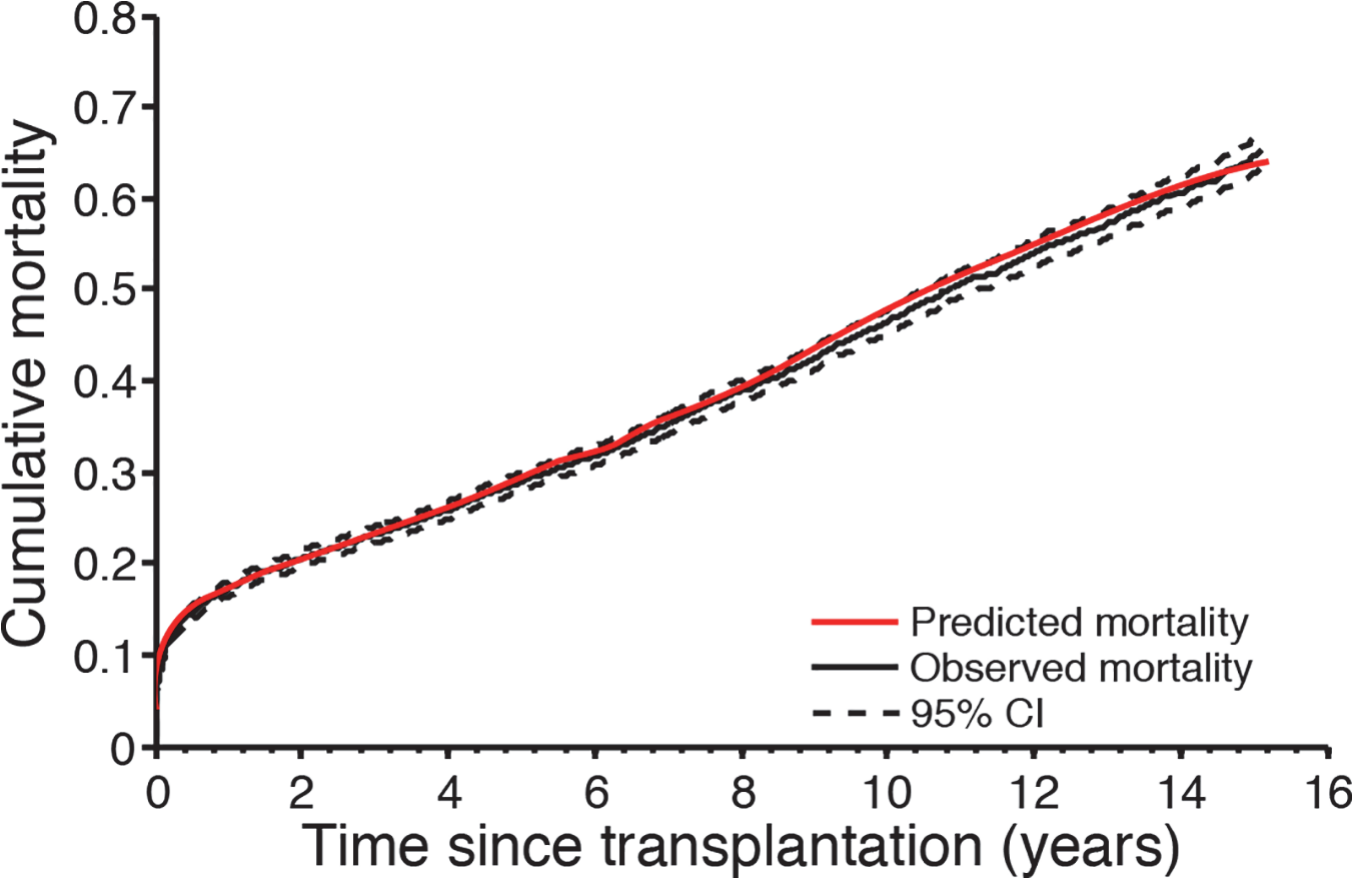
Cumulative mortality for the internal validation cohort (IVC). The black solid line shows the observed cumulative mortality and dotted lines show the 95% confidence interval (estimated with Kaplan-Meier failure function) in the IVC. The red solid line shows the predicted survival for transplanted patients in the IVC.

As illustrated in Fig. 2, the predicted cumulative failure for patients in the IVC stayed, with a single exception at 10 years, within the 95% confidence limits for the KM estimate, indicating good calibration. The predicted versus actual 1-year, 5-year, and 10-year survival rates were 83.7% versus 82.6%, and 71.4% versus 70.8%, and 54.8% versus 54.3%, respectively in the DC; 83.7% versus 82.8%, and 71.5% versus 71.5%, and 54.9% versus 53.8%, in the IVC; and 84.5% versus 84.4%, 72.9% versus 75.6%, and 57.5% versus 57.5% in the NTTD.

The discrimination (C-index) for survival in IVC was 0.592 (95% CI: 0.582–0.602) and was not statistically significantly different from that of the DC (p = 0.170). The C-index of patients transplanted in the later time era (TVC) was 0.645 (95% CI: 0.633–0.657) and for the NTTD it was 0.590 (95% CI: 0.562–0.617). The AUROC for the one year mortality in the IVC was 0.62 (95% CI: 0.606–0.638), which was significantly less than for the DC (p = 0.002), whereas it was not significantly different from that for TVC and NTTD (p = 0.466 and p = 0.408, respectively).

### ITHSA versus DRI, IMPACT, and RSS

As shown in Table 5, the C-index for the IHTSA was significantly better than the DRI, IMPACT, and RSS models in the DC and IVC (p<0.001), and in the NTTD (p<0.05). The IHTSA model also had a significantly higher discrimination (AUROC) for one-year mortality in DC and IVC (p<0.001), as shown in Table 6. The Hosmer-Lemeshow (HL) chi-square in IVC was 11.7 for the IHTSA model (p = 0.166), suggesting that there was good calibration. However, the calibration for the RSS and IMPACT models was poor (0.026 and 0.038, respectively).

**Table 5:** Comparison of C-index between different risk models used to predict overall survival.

	C-index (95% CI)			
	DC	IVC	TVC	NTTD
	n = 41,780	n = 8,569	n = 6,276	n = 1,285
IHTSA	0.60 (0.59–0.60)	0.59 (0.58–0.60)	0.65 (0.63–0.66)	0.59 (0.56–0.62)
DRI	0.54 (0.54–0.55)^†^	0.54 (0.53–0.55)^†^	0.58 (0.56–0.60)^†^	0.55 (0.52–0.58)*
IMPACT	0.56 (0.55–0.57)^†^	0.55 (0.54–0.56)^†^	0.63 (0.61–0.65)	0.52 (0.49–0.56)^†^
RSS	0.56 (0.56–0.57)^†^	0.56 (0.55–0.57)^†^	0.64 (0.62–0.65)	0.54 (0.51–0.58)*

*p < 0.05

^†^p ≤ 0.001 compared with ITHSA. CI, confidence interval; DC, derivation cohort; DRI, donor risk index for transplantation (reported in the paper by Weiss et al. [6]); IMPACT, index for mortality prediction after cardiac transplantation (reported in the paper by Weiss et al. [7]); ITHSA, international heart transplantation survival algorithm; IVC, internal validation cohort; NTTD, Nordic thoracic transplantation database; RSS, risk-stratification score (reported in the paper by Hong et al. [9]); TVC, temporal validation cohort.

**Table 6:** Comparison of AUROCs between different risk models used to predict one- year mortality.

	AUROC (95% CI)			
	DC	IVC	TVC	NTTD
	n = 40,117	n = 8,244	n = 4,248	n = 1,194
IHTSA	0.65 (0.64–0.66)	0.62 (0.61–0.64)	0.64 (0.62–0.67)	0.59 (0.47–0.72)
DRI	0.56 (0.56–0.57)^†^	0.56 (0.54–0.57)^†^	0.59 (0.57–0.61)^†^	0.55 (0.43–0.68)
IMPACT	0.61 (0.60–0.61)^†^	0.59 (0.58–0.61)^†^	0.65 (0.63–0.67)	0.52 (0.38–0.66)
RSS	0.61 (0.61–0.62)^†^	0.60 (0.59–0.62)*	0.66 (0.64–0.68)	0.58 (0.45–0.71)

*p < 0.05

^†^p < 0.001 compared with ITHSA. AUROC, area under the receiver operating curve; CI, confidence interval; DC, derivation cohort; DRI, donor risk index for transplantation (reported in the paper by Weiss et al. [6]); IMPACT, index for mortality prediction after cardiac transplantation (reported in the paper by Weiss et al. [7]); ITHSA, international heart transplantation survival algorithm; IVC, internal validation cohort; NTTD, Nordic thoracic transplantation database; RSS, risk stratification score (reported in the paper by Hong et al. [9]); TVC, temporal validation cohort.

### Time-dependent hazard ratio

As shown in Tables 3 and 4, the time-dependent hazard ratio (tHR) varied over time. The tHR for donor age declined by 8%, and for the recipient age it increased by 13%. In a subgroup analysis, the tHR for a 30 years old or younger recipient was constant the first 5 years post transplant in contest to a 60 years old or older recipient, where it increased with 6%. Although, the tHR decline for donor age was similar in the different subgroups, Table 5. The largest change in tHR (a 43% decline) was found for patients treated with extracorporeal membrane oxygenation (ECMO) prior to transplantation. Patients who were previously transplanted had the largest hazard ratio at one year (tHR = 1.59), followed by mechanical ventilation prior to transplant (tHR = 1.24). Interestingly, our analyses showed that the tHR for duration of ischemia declined to 1.0 after 10 years.

### Sub-group analysis

**Fig 3:**
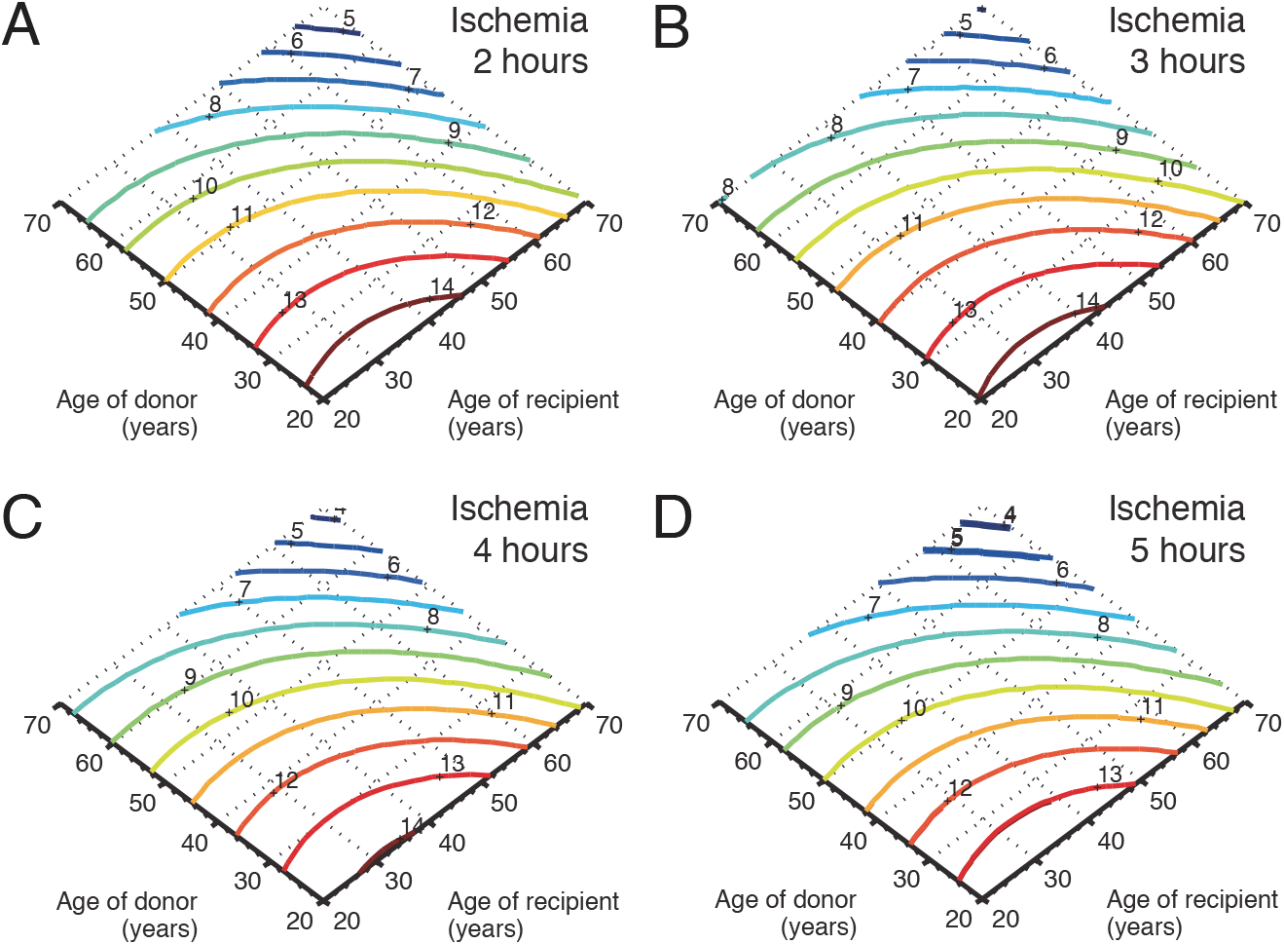
Influence of age and duration of ischemia on survival. Panel (A) illustrates, in matched cohorts, the influence of donor age and recipient age on survival when the duration of ischemia is fixed to 2 hours, and panels B, C, and D illustrate the influence on survival at 3, 4, and 5 hours of ischemia, respectively. The colored solid lines show the predicted survival in years (dark blue: worst; dark red: best).

**Fig 4:**
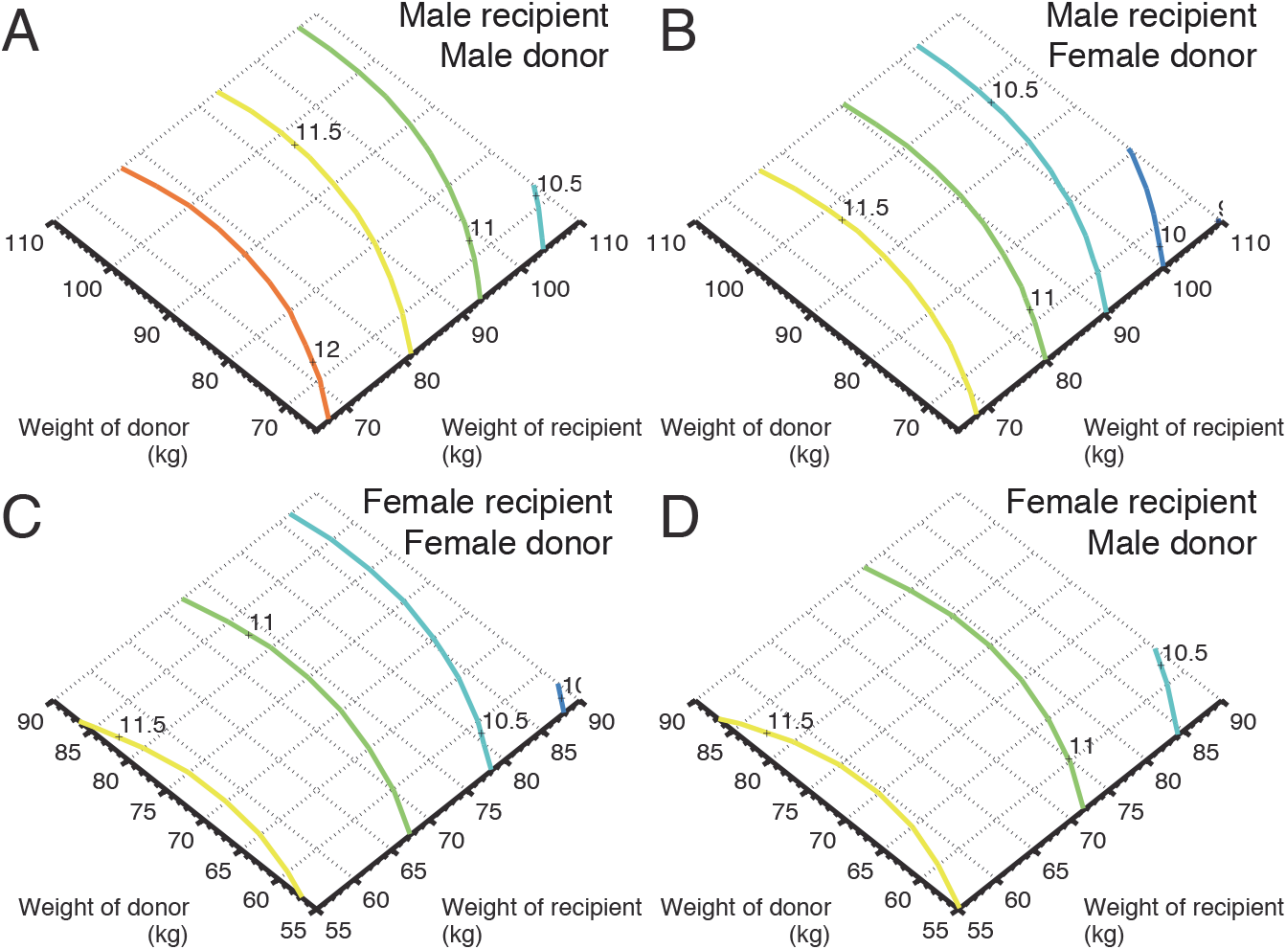
Influence of weight and gender match. Panel (A) illustrates, in matched cohorts, the influence of donor weight and recipient weight on survival for male-male donor-recipient pairs, and panels B, C, and D illustrate the influence on survival for female-male, female-female, and male-female donor-recipient pairs, respectively. The colored solid lines show the predicted survival in years (dark blue: worst; dark red: best).

As illustrated in Fig. 3, in the matched cohorts, an increase in donor age from 25 to 60 years decline the estimated median survival time with 4 years (from 14 to 10 years), and an increase in the recipient age from 25 to 60 years imply that the estimated median survival time decline with 2 years. Furthermore, an increase in duration of ischemia have a limited impact on survival time and the duration of ischemia does not seem to be influenced by the donor age. A similar analysis using the donor weight, recipient weight and gender match showed that female donors matched to male recipients had in average 7 months reduced expected median survival time. For female recipients there was no difference in estimated survival depending on gender match, Fig. 4.

### Quantification of survival years using CART

**Fig 5:**
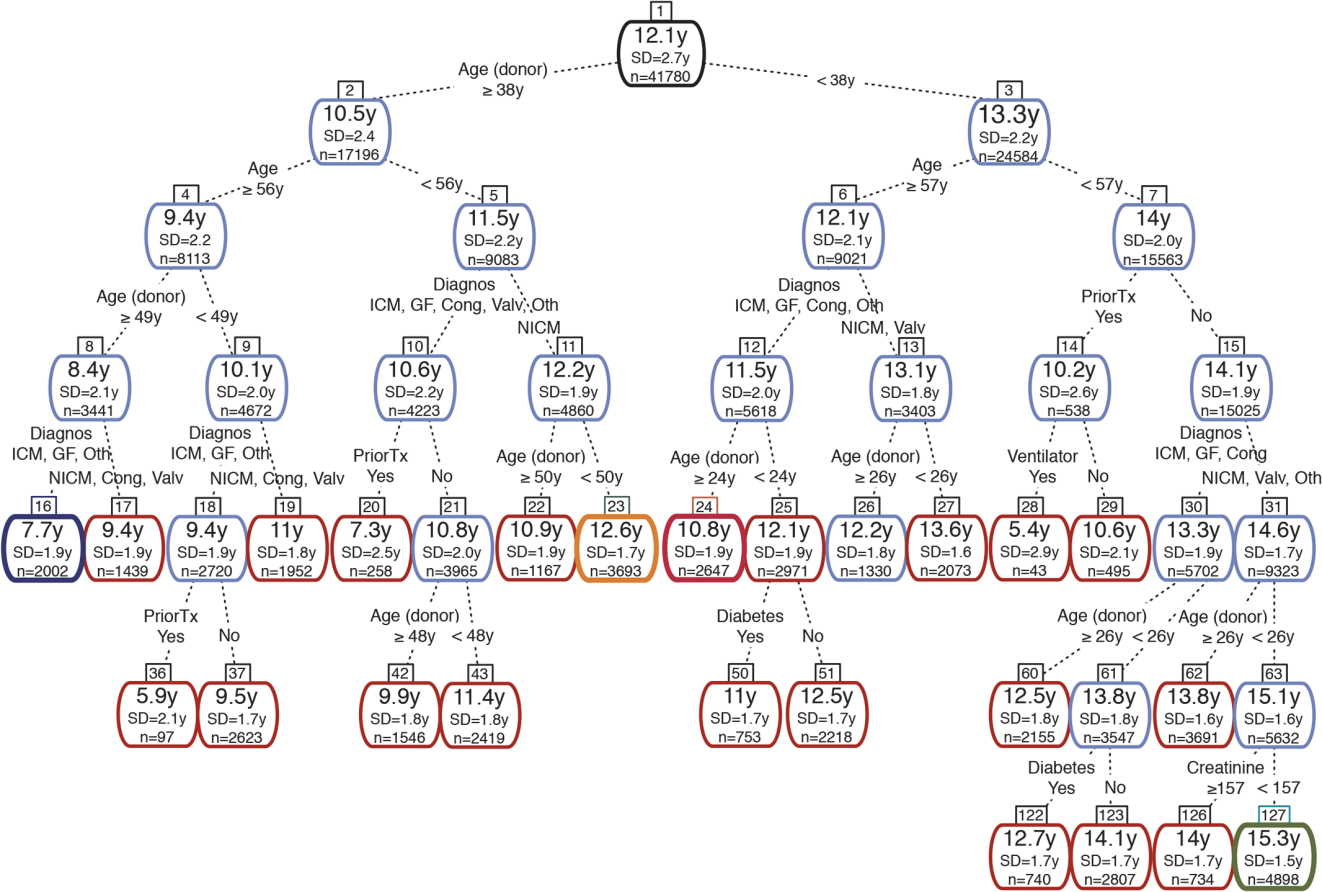
Relative importance for the IHTSA variables. The numbers within the nodes and leaves represent the predicted median survival together with standard deviation (SD) in years (y), and the number of patients the results is based on (n). The decision variable and the criterion are shown on the dotted lines between the nodes. The most important variable is the top node. Light blue coloring of the nodes shows an internal node and red represents a leaf node. Dark blue, dark red, yellow, and green coloring represent the four leaf nodes for which the observed cumulative mortality is plotted against time in Fig. 6. The tree has been pruned to a cost-complexity parameter of 0.0035. Cong, congenital; ICM, ischemic cardiomyopathy; NICM, non-ischemic cardiomyopathy; GF, graft failure; Valv, valvular; Oth, other.

**Fig 6:**
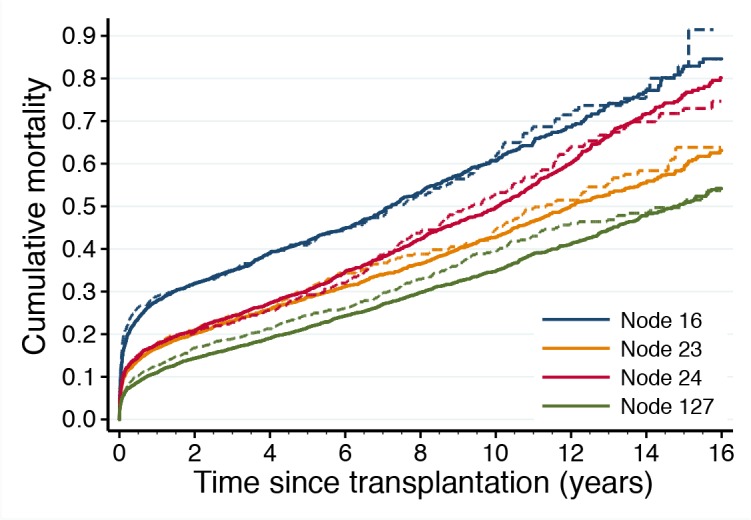
Validation of the IHTSA variable decision tree. This graph illustrates the difference in observed cumulative mortality for patients belonging to leaf nodes 16, 23, 24, and 127 in the decision tree. The line color corresponds to the leaf node color in Fig. 5. The solid lines show the observed cumulative mortality for transplanted patients in the derivation cohort and dashed lines show the observed cumulative mortality for transplanted patients in the internal validation cohort (estimated with Kaplan-Meier failure function).

Of the 43 top-ranked variables included in the IHTSA model, only seven remained in the pruned decision tree. As illustrated in Fig. 5, the top-ranked variable, donor age, split the age at 38 years. Recipients matched to a donor younger than 38 years had an additional expected median survival time of 2.8 years. The relative importance of the donor age was 46%, followed by recipient age (18%), diagnosis (15%), donor cause of death (10%), previous transplant (6%), donor gender (3%), diabetes (1.4%), and treatment with mechanical ventilator prior to transplantation (0.5%). Duration of ischemia, body size and blood group were found to be of minor importance. Patients who had a previous transplant and those in need of a mechanical ventilator had the worse prognosis irrespectively of the age of the donor or recipient. The CART analysis identified three cut-offs for donor age—25 year, 38 years, and 50 years—but only one cut-off for recipient age, 57 years. The decision tree was validated by estimating the Kaplan Meier survival for the patients belonging to leaf node 16, 23, 24, and 127. As illustrated in Fig. 6, there was no significantly difference in survival for the DC and IVC in the four groups (p = 0.759, p = 0.861, p = 0.612, p = 0.214, log-rank test), and the trend was concordant with the decision tree.

### Practical Calculation of the Estimated Survival

**Fig 7:**
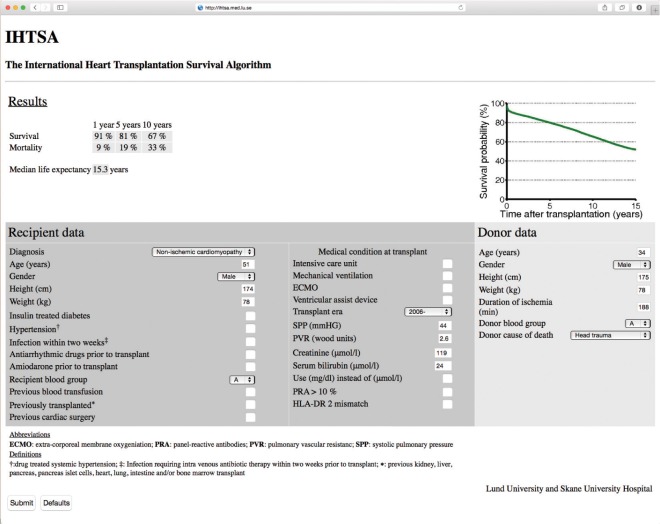
The International Heart Transplantation Survival Algorithm (IHTSA) as a web application. The IHTSA has been implemented as an interactive program that estimates median, 1-, 5-, and 10-year survival, and the benefit of adding (or removing) properties from an individual recipient and the potential donor (http://www.ihtsa.med.lu.se). ECMO, extracorporeal membrane oxygenation; HLA, human leukocyte antigen; PRA, panel reactive antibody; PVR, pulmonary vascular resistance; SPP, systolic pulmonary pressure. ^†^Drug treated systemic hypertension. ‡Infection requiring intra venous antibiotic therapy within two weeks prior to transplant. *Previous transplant——previous kidney, liver, pancreas, pancreas islet cells, heart, lung, intestine and/or bone marrow transplant.

It is almost impossible to compute the estimated survival using a machine-learning model such as ANN, by hand. A Web-based calculator (http://www.ihtsa.med.lu.se) has been developed as an interactive web-application that estimates median, 1-, 5-, and 10-year survival, and the effect on survival by adding (or removing) risk factors from an individual recipient and its potential donor using our ANN survival model, Fig. 7.

### Organ allocation simulation

**Fig 8:**
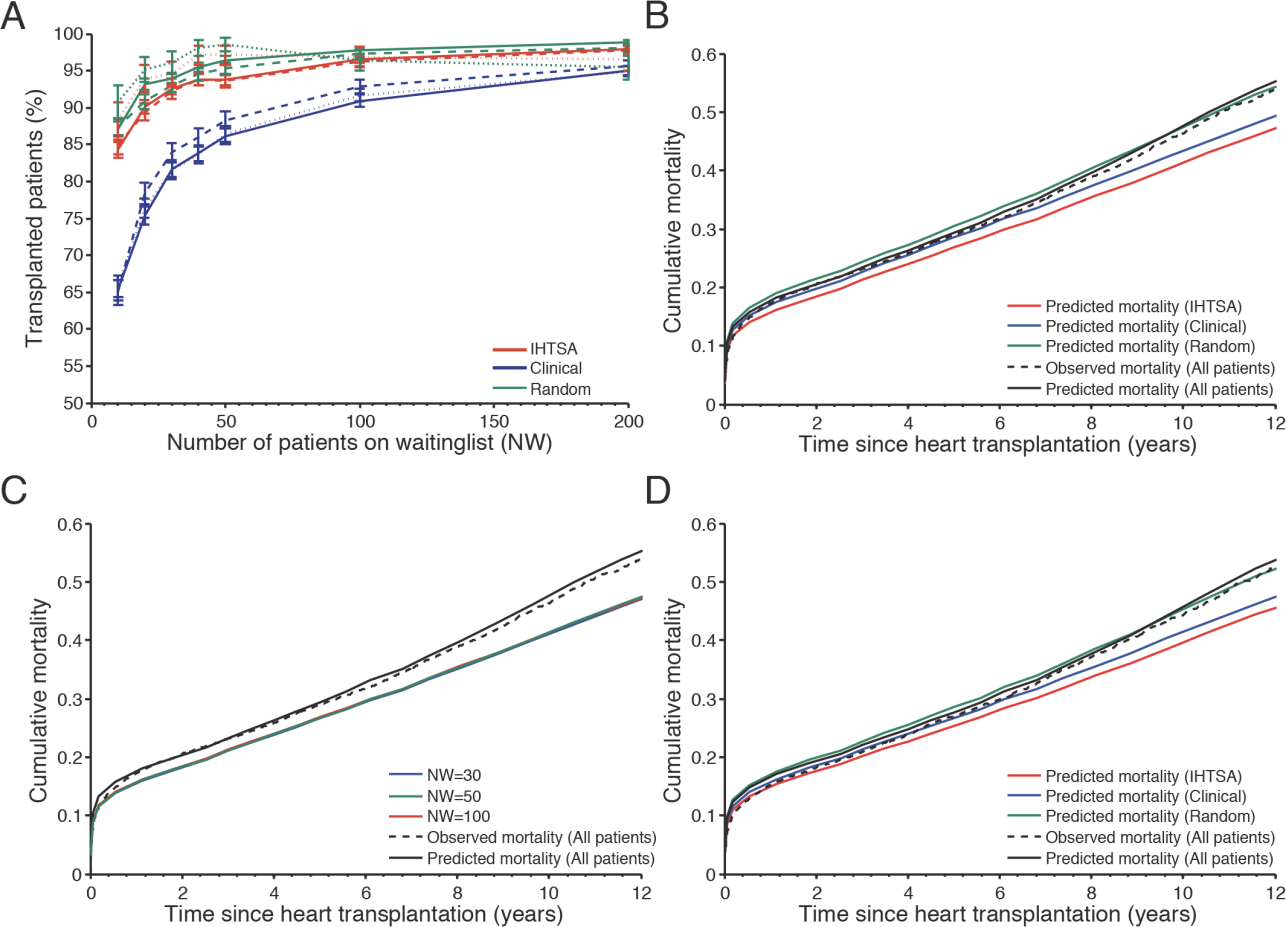
Predicted cumulative mortality for different organ allocation models. In panel (A) the graph shows the number of transplanted patients for The IHTSA model (red lines), Clinical model (blue lines) and Control (green lines) influenced by the size of the waiting list. The internal validation cohort (IVC) is presented with solid lines, temporal validation cohort (TVC) dashed lines and external validation cohort NTTD dotted lines. In panel (B) the graph shows the difference in predicted cumulative mortality as a function of time since heart transplantation for a waiting list including 50 patients in the IVC (N = 8,569). The solid black lines present the observed mortality and the dotted lines the predicted mortality for all patients. Panel (C) shows the difference in predicted cumulative mortality for IHTSA model influenced by the number of patients on the waiting list (NW). In panel (D) the results from the sub analysis including patients from the IVC, who were not treated in the intensive care unit and were not on life support prior to transplantation, are presented (N = 4,868).

As illustrated in Fig. 8A, the IHTSA model succeeded to allocate more organs (3%-19%, depending on the size of the waiting list), compared with the clinical model, p<0.0001. When the waiting list included 50 patients or more, the increase of possible transplants was marginal for the IHTSA model. The Clinical model reaches the same level when the waiting list included 200 patients, S3A Table. The largest difference in allocated organs could be seen in the NTTD cohort when a small waiting list were used, NW = 10 patients. The IHTSA mode allocated 22% more organ compared with the Clinical model, S3B Table. The estimated median survival time was 6–9 months longer for the IHTSA compared with the clinical model, p<0.001, Fig. 8B. The average improvement in survival compared with the control group (randomized allocation) was 34 months for the IHTSA model, p<0.001, S3A Table. The size of the waiting list did not effect the survival, Fig. 8C.

Patients on life support have a high mortality risk post transplant and probably the largest mortality risk on the waiting list. To analyze which bias this could introduce to the results for the IHTSA model, a sensitivity analysis were performed (N = 4,868) where the high-risk candidates (treated in ICU or on life support prior to transplantation) were excluded from the analysis. As illustrated in Fig. 8A and D, the IHTSA demonstrated a similar increase in the number of allocated organs and predicted survival time compared with the Clinical model.

## Discussion

In this paper, we present the first international survival prediction model for heart-transplanted patients. By using such a model, we can make an improved outcome prediction based on the characteristics of both the recipient and donor. The findings from this study show that a flexible non-linear ANN model can be used to predict both short- and long-term mortality with higher accuracy. Furthermore, we demonstrate that a survival prediction based organ sharing system may allocate more organs compared with a criterion-based system.

The registry used to develop the model is unique in many ways. The registry has been collected data since the 1980’s, it provide data from more than 300 participating institutions and include data from almost 70% of all heart transplantations performed in the world. The most essential variable in our model was donor age, which is in consistence with previous findings [21]. However, we further show that donor age influences the estimated survivals by a factor of two compared with the recipient age and that recipients matched to a donor younger than 38 years had an additional expected median survival time of three years. Previous single center reports, have suggested that it is more beneficial in terms of patient survival to receive an allograft from a donor >40 years old than to remain on the waiting list [4,22]. Our finding raises the question again; may it be more beneficial in terms of patient survival to remain on the waiting list than to receive an allograft from an older donor, especially for non-urgent status-II patients?

Prolonged duration of ischemia has an adverse effect on survival [5,23,24]. Interestingly, our analyses showed that the duration of ischemia affects the discrimination of survival less than expected. The reason might be that our model was developed to predict overall survival and previous studies have been focused on short-term survival. Our finding that the HR was most prominent in the early postoperative era but declined over time supports this.

The influences of body size and gender match are more controversial than duration of ischemia. The "classic rule” from the early 1990s, supported by previous studies, requires that the donor/recipient weight ratio is not less than 0.8 [4,25]. In the present study, the minor importance of body size match is more consistent with recently published studies [26]. However, we show that a female donor matched to a male recipient has a detrimental effect on survival due to the donor gender and not the body size. A gender effect on graft survival has been reported in renal and liver transplantation but also observed for cardiac allografts, one might speculate that the differences in immunogenicity according to donor gender may be a possible explanation [27].

As illustrated in the sub-group analysis with matched cohorts, we could identify both a non-linear relationship and interactions between the recipient and donor risk factors. For example the HR for a 30 years old recipient were constant the first 5 years post transplant in contest to a 60 years old recipient where it increased with 6%. Furthermore, the HR for different donor age groups was not consistent. The strength of the ANN model is its flexibility to model non-linear relationships and all possible interactions in a virtual fashion, and furthermore it is not dependent on the assumption of a proportional hazard [21,28]. In the present study, the hazard ratio varied over time in several of the variables. A methodology based on standard linear models such as Cox proportion hazard regression requires *a priori* knowledge of the variable relationships and that the hazards are proportional, and thus not optimal to use in the present material.

To gain acceptance for a model, it is important to quantify the model’s generalizability. By using validation cohorts from an external database and a different time era, the generalizability for the model will be evaluated in an accurate way. The IHTSA model was validated on an internal validation cohort, a temporal validation cohort, and on a regional dataset. Validation in a similar way has only been performed in one of the risk-stratification models compared in this study, the IMPACT model [29]. However, that study did not show whether there was a difference in discrimination between the different cohorts. Our ITHSA model presents a similar degree of discrimination in all of the validation cohorts.

Even if comparison of risk models remains controversial, the C-index is probably the best statistical tool for describing performance [30,31]. A C-index of 0.65 may appear to be low but it should be kept in mind that the IHTSA model predicts long term survival, up to 18 years post transplant and it is higher than previously reported. Despite the fact that the DRI, IMPACT, and RSS were designed to predict one-year mortality, the IHTSA shows superior or similar discrimination in all of the cohorts. Furthermore, the new model generated a unique survival prediction for almost all of the patients. Given that the model in our study was optimized for good prediction at all time points (and not at any specific time point), this demonstrates high flexibility of the IHTSA model.

A model including 43 variables (32 recipient and 11 donor variables) where advanced machine learning algorithms are used makes it impractical or impossible for computation by hand. By developing a model that can simulate the outcome for a patient undergoing heart transplantation, we will be able to avoid poor case match and identify best possible match in the clinical setting. Our web-based calculator (http://www.ihtsa.med.lu.se) allows the user to perform convenient interactive calculations of estimated survival and also of the predicted effects on survival of adding (or changing) different patient characteristics or use of devices together with a donor’s specific profile. The estimate of median life-years in the web-application is provided to illustrate the potential change in long-term mortality with the addition of recipient and/or donor risk factors; however, the potential error for such long-term estimates may be large for low-risk patients.

During 2011, approximately 17 000 donors were reported [32]. Unfortunately, not more than one third of all donors could be utilized for heart transplantation. One explanation for this may be an uncertainty in the risk of early and late graft dysfunction, which means that some suitable donors are not accepted. Although there are many donor predictors of allograft discard in the current era, these characteristics seem to have little effect on recipient outcomes when the hearts are transplanted [33]. A more liberal use of cardiac allografts with relative contraindications may be warranted. However, predicting the impact of a proposed new organ allocation policy is difficult since there are many factors that can change in unpredictable ways when rules change [34]. In the present study, a simulation model evaluated the influence on such different allocation policy. The results show that the survival predicting model (IHTSA) increase the number of organ that could be used without compromise the survival time compared with a traditional criterion based model. Furthermore, the number of possible matches increases by the size of the waiting list, which support the use of centralized organ allocation system such as United Network for Organ Sharing (UNOS).

The waiting lists used worldwide typically consist of a limited number of patients. Since that is not about to change in the near future, the IHTSA model can be used as a virtual recipient-donor matching tool that models survival for potential recipients on a waiting list when a donor’s heart is available. The program then produces a ranking list; from low-risk to high-risk recipients, that can be presented to the transplant physicians [35].

The results of this study carry limitations associated with the retrospective analysis of a registry database, the quality of the source data, the number of missing data, and the lack of standardization associated with multicenter studies (such as different immunosuppressive regimens and different matching criteria), as has been described previously [36]. Some data elements, including center volume and geographic location, were not released by the ISHLT registry. Duration on the waiting list, deaths pre-listing and deaths on the waiting list would have been desirable to include in the model. Unfortunately, the registry does not collect these data. However, with the improvement of heart failure treatment and mechanical assist devices, the waiting-list mortality has decreased and the post transplant outcome has become more significant [9,37–40]. The predicted survival in this study did not account for the race mismatch and may have underestimated the mortality rates in these cases. A small percentage of the derivation cohort included patients from the NTTD which may have had a minor effect on the validation results.

### Conclusions

We introduce the first international post-heart transplantation survival prediction model, the IHTSA. Furthermore, we present a novel approach of how to analyze and validate the models influence on the clinical practice using simulation techniques. By combining machine-learning approaches, we could quantify the number of years gained and the relative importance of recipient and donor risk factors. The IHTSA web-application may be useful for providing estimation of survival at the patient-level after transplantation. This might further serve as a virtual matching tool, making us more confident in the post-transplantation performance, which may improve survival and maximize the number of organs that can be used.

## Supporting Information

S1 Methods Survival model. Extended description of the survival analysis using neural networks, including the calibration and validation of neural networks, and the risk variable identification.
(PDF)

S1 Table Complete listing of the baseline characteristic. A) Recipient demographic and clinical characteristic of the study population. B) Recipient hemodynamic, laboratory and urgency characteristic of the study population. C) Donor demographic and characteristic of the study population. D) Blood group, immunology and era of the study population.
(PDF)

S2 Table Time-dependent hazard ratios for different subgroups. (PDF)

S3 Table Organ allocation simulation. Influences of the allocated organs, and the predicted and observed survival time depending on the waiting list size, using the IHTSA model, Clinical model respectively by random. A) Applied on the internal validation cohort. B) Applied on the external validation cohort, Nordic Thoracic Transplantation Database.
(PDF)
