# Supplementary material for: The International Heart Transplant Survival Algorithm (IHTSA): A New Model to Improve Organ Sharing and Survival
Source: PLoS One. 2015 Mar 11;10(3):e0118644. doi: 10.1371/journal.pone.0118644 (PMC4356583; doi:10.1371/journal.pone.0118644)
Supplement: S1 Methods — Extended description of the survival analysis using neural networks, including the calibration and validation of neural networks, and the risk variable identification. (PDF) [file pone.0118644.s001.pdf]

S1 Methods. Survival model.

Survival analysis using neural networks

Cox regression models are the most commonly used methods for survival analysis in clinical research. Given its assumptions of proportionality of the hazard and the usual linear modelling of the covariates one would like to extend the analysis methods. Models using ANN have the ability to model the hazard with explicit time dependency and flexible nonlinear effects among the covariates. Biganzoli et al. showed that by treating the time interval as an input variable in a standard feed-forward ANN with a cross-entropy error function, it was possible to estimate smoothed discrete hazards as conditional probabilities of failure [1]. The survival models used in this project follows the principles described in Biganzoli et al. with the extension of using ensembles of ANNs instead of a single one [2]. For a general introduction to ANN see the Cross et al [3]. The ANNs were implemented as feed-forward multilayer perceptrons (MLP) with one hidden layer with the hyperbolic tangent as the activation function. The following error function was used during the training of the ANN models,

$$E = \sum_i \sum_l \left[d_{il} \log\left(h_l(x_i, a_l)\right) + (1 - d_{il}) \log\left(1 - h(x_i, h_l)\right)\right]$$

where $h_l(x_i, a_l)$ is a smoothed estimate of the discrete hazard function for time interval l, which is modeled by the ANN output. The variables (x_i, a_l) represents the covariates for patient i and midpoint time for interval l, respectively. The event indicator d_{il} variable is one if uncensored patient i has the event in time interval l and zero otherwise. In order to have the possibility to regularize the ANN models a weight decay term was added to the above error function. This term, $E_r = \alpha \sum_j \omega_j^2$ introduces the parameter α , which is tuned during the model calibration procedure (see below). Finally to optimize the performance of the ANN model an ensemble approach were used, where several ANNs were combined into a single prediction model. The output of the ANN ensemble was computed as the mean of the output of the individual members in the ensemble. The ensemble was constructed by training the ANNs on different training sets, obtained from the random imputation technique when dealing with missing data. The ensemble size was 10 and no effort was used to optimize this number. Given the discrete hazard function $h_l(x_i, a_l)$ and the definition $S(t_0) = 1$ the full survival curve can be constructed according to,

$$S(t) = \prod_{l:t_l<t} (1 - h_l)$$

where t_l is the end time for interval l.

Calibration and validation of the ANN models

Calibration of each individual ANN was accomplished by minimizing the above error function using resilient back-propagation. To find the optimal regularization parameter and the optimal number of hidden nodes for the ANN 5-fold cross-validation was utilized. The number of hidden nodes was determined based on experiments starting with a single node and increasing the number of nodes until the highest accuracy was found for the validation sets. By a similar procedure the α -parameter was chosen to optimize the validation performance. When all parameters were set a new calibration using the full training dataset was performed. Throughout the model calibration the ensemble approach was used utilizing 10 different datasets, obtained from the missing data imputation technique (see below). The derivation cohort was used to calibrate and identify the optimal architecture for the ANN.

Risk variables identifications

To identify important risk variables and to select the optimal set of risk variables used in the survival model, a ranking of risk variables was performed [4,5]. A baseline C-index is created using all variables. The ranking list was then obtained by measuring the change of the C-index, as compared to the baseline, when a risk variable was excluded from the model. The highest ranked variable corresponds to the largest decrease of the C-index when it is excluded from the model. The lowest ranked variable will have the smallest effect on the C-index when excluded from the model and was subsequently be removed from the model. A new survival model was created and a new baseline C-index was computed, giving a new ranking list from which the lowest ranked variable again was removed. This backward elimination procedure was repeated until only one variable was left. The order in which the variables were removed constituted the final ranking list. Throughout this procedure full calibration of the model was performed, see Figure 1A for an illustration of the procedure including both model calibration and risk variable identification. The selection of the final set of variables was based in the obtained ranking list and was selected when no performance increase was found when adding the next variable from the ranking list. This resulted in a final model including 43 inputs, 18 hidden nodes, 25 time intervals and 10 committee members in the ensemble.

References

1. Biganzoli E, Boracchi P, Mariani L, Marubini E (1998) Feed forward neural networks for the analysis of censored survival data: a partial logistic regression approach. Stat Med 17: 1169- 1186.
2. Hansen LK, Salamon P (1990) Neural network ensembles. IEEE Transactions on Pattern Analysis and Machine Intelligence 12: 993-1001.
3. Cross SS, Harrison RF, Kennedy RL (1995) Introduction to neural networks. Lancet 346: 1075-1079.
4. Nilsson J, Ohlsson M, Thulin L, Höglund P, Nashef SAM, et al. (2006) Risk factor identification and mortality prediction in cardiac surgery using artificial neural networks. J Thorac Cardiovasc Surg 132:12-19.
5. Carlsson A, Wingren C, Kristensson M, Rose C, Fernö M, et al. (2011) Molecular serum portraits in patients with primary breast cancer predict the development of distant metastases. Proceedings of theNational Academy of Sciences of the United States of America: 1-6.
