# Supplementary material for: The International Heart Transplant Survival Algorithm (IHTSA): A New Model to Improve Organ Sharing and Survival
Source: PLoS One. 2015 Mar 11;10(3):e0118644. doi: 10.1371/journal.pone.0118644 (PMC4356583; doi:10.1371/journal.pone.0118644)
Supplement: S1 Table — A) Recipient demographic and clinical characteristic of the study population. B) Recipient hemodynamic, laboratory and urgency characteristic of the study population. C) Donor demographic and characteristic of the study population. D) Blood group, immunology and era of the study population. (PDF) [file pone.0118644.s002.pdf]

S1A Table. Recipient demographic and clinical characteristics of the study population from ISHLT and the Nordic Thoracic Transplantation database.

		ISHLT			NTTD
		Derivation cohort	IVC	TVC	
	N	N = 41,780	N = 8,569	N = 6,276	N = 1,285
Recipient's diagnosis	57,863				
Ischemic cardiomyopathy		17,670 (42%)	3,630 (42%)	2,166 (35%)	450 (36%)
Non-ischemic cardiomyopathy		19,265 (46%)	3,946 (46%)	3,213 (51%)	613 (50%)
Congenital		867 (2%)	180 (2%)	203 (3%)	49 (4%)
Graft failure		846 (2%)	168 (2%)	151 (2%)	5 (0%)
Valvular heart disease		1,254 (3%)	266 (3%)	130 (2%)	38 (3%)
Miscellaneous		1,878 (4%)	379 (4%)	413 (7%)	83 (7%)
Age (year)	57,910	51±12	51±12	51±13	50±11
Female gender	57,910	8,463 (20%)	1,827 (21%)	1,485 (24%)	286 (22%)
Height (cm)	45,100	173±9·4	173±9·5	174±9·6	175±8·6
Weight (kg)	45,464	78±16	78±16	81±17	77±14
History of cigarette use	15,299	3,604 (42%)	760 (41%)	1,896 (46%)	83 (12%)
COPD	31,135	789 (3%)	142 (3%)	138 (4%)	36 (5%)
Diabetes	33,471	4,802 (20%)	1,006 (21%)	1,059 (25%)	82 (11%)
History of peptic ulcer	32,833	1,102 (5%)	221 (4%)	75 (2%)	30 (4%)
Hypertension[*]	33,613	9,185 (38%)	1,904 (38%)	1,526 (44%)	141 (19%)
Peripheral vascular disease	33,535	833 (3%)	182 (4%)	102 (3%)	20 (3%)
History of cerebrovascular disease	32,387	916 (4%)	197 (4%)	171 (4%)	
Cerebrovascular event	30,080	550 (3%)	127 (3%)	103 (3%)	45 (6%)
Dialysis pre-transplant	34,908	322 (1%)	77 (1%)	89 (2%)	15 (2%)
Infection within two weeks[†]	32,624	2,326 (10%)	493 (10%)	410 (10%)	40 (5%)
Cytomegalovirus status	20,776	10,019 (74%)	2,106 (74%)	2,291 (71%)	778 (67%)
Antiarrhythmic drugs prior to transplant	31,660	8,472 (37%)	1,755 (37%)	1,255 (39%)	263 (36%)
Amiodarone prior to transplant	31,114	5,787 (26%)	1,223 (26%)	993 (32%)	172 (23%)
Implantable defibrillator	28,675	9,142 (44%)	1,934 (45%)	2,921 (93%)	182 (24%)
Unstable angina	23,133	2,336 (14%)	503 (15%)	69 (3%)	70 (9%)
PRA > 10%	31,590	1,841 (8%)	370 (8%)	402 (11%)	87 (7%)
Previous blood transfusion	19,138	7,574 (51%)	1,569 (51%)	966 (86%)	
Previous transplanted[#]	54,001	1,160 (3%)	224 (3%)	200 (3%)	5 (0%)
Previous cardiac surgery	34,004	6,594 (28%)	1,371 (28%)	2,465 (60%)	305 (24%)
Malignancy prior to transplantation	36,176	1,014 (4%)	221 (4%)	310 (7%)	
Oxygen consumption at exercise	7,216	11·8±3·1	11·7±3·2	11·9±3·2	
Activities of daily living	10,184				
No assistance		2,319 (28%)	488 (29%)	58 (20%)	
Some assistance		4,528 (55%)	887 (53%)	132 (46%)	
Total assistance		670 (8%)	164 (10%)	42 (15%)	
Working for income	12,639	414 (6%)	82 (5%)	232 (6%)	

Data are mean (SD), number (%). The numbers are calculated on patients with available data. [*]Drug treated systemic hypertension. [†]Infection requiring intra venous antibiotic therapy within two weeks prior to transplant. [#]Previous transplant — previous kidney, liver, pancreas, pancreas islet cells, heart, lung, intestine and/or bone marrow transplant. COPD, chronic obstructive pulmonary disease; ISHLT, International Society for Heart and Lung Transplantation; IVC, internal validation cohort; NTTD, Nordic Thoracic Transplatation Database; PRA, panel reactive antibody; TVC, temporal validation cohort.

S1B Table. Recipient hemodynamic, laboratory and urgency characteristics of the study pop-ulation from ISHLT and the Nordic Thoracic Transplantation Database.

		ISHLT			NTTD
		**Derivation cohort**	**IVC**	**TVC**	
	N	N = 41,780	N = 8,569	N = 6,276	N = 1,285
PVR (wood units)	27,053	2·6±1·9	2·6±2·1	2·5±1·6	3·0±1·8
SPP (mmHg)	31,226	44±15	44±16	43±15	
Creatinine (µmol/l)	34,017	120± 64	120±67	117±66	111±59
Serum bilirubin (mg/dl)	31,020	1·43±3·54	1·29±2·37	1·20±2·05	1·31±0·91
Serum albumin (g/l)	17,583	36·5± 7·7	36·5± 7·5	36·7±7·0	
Medical condition at transplant	35,172				
Not hospitalized		11,374 (45%)	2,338 (45%)	2,308 (57%)	590 (76%)
Hospitalized		4,106 (16%)	887 (17%)	669 (17%)	92 (12%)
ICU		9,679 (38%)	1,989 (38%)	1,043 (26%)	97 (12%)
Inotropic support prior to transplant	34,271	12,136 (49%)	2,627 (51%)	1,561 (38%)	
Mechanical ventilation	32,712	731 (3%)	151 (3%)	138 (3%)	
Intra-aortic balloon pump	33,460	2,160 (9%)	456 (9%)	332 (8%)	
ECMO	32,836	106 (0%)	23 (0%)	39 (1%)	
Ventricular assist device	24,509	4,367 (26%)	902 (26%)	1,429 (34%)	

Data are mean (SD) or number (%). The numbers are calculated on patients with available data. ECMO, extracorporeal membrane oxygenation; ICU, intensive care unit; ISHLT, International Society for Heart and Lung Transplantation; IVC, internal validation cohort; NTTD, Nordic Thoracic Transplatation Database; PVR, pulmonary vascular resistance; SPP, systolic pulmonary pressure; TVC, temporal validation cohort.

S1C Table. Donor demographic and characteristics of the study population from ISHLT and the Nordic Thoracic Transplantation Database.

		ISHLT			NTTD
	N	Derivation cohort<br>N = 41,780	IVC<br>N = 8,569	TVC<br>N = 6,276	N = 1,285
Age (year)	57,910	34±13	34±13	35±13	39±13
Female gender	57,910	13,011 (31%)	2,657 (31%)	1,981 (32%)	451 (35%)
Height (cm)	48,782	174·8±9·4	174·8± 9·3	174·7±9·4	176·7±8·6
Weight (kg)	50,097	78±16	78±17	81±18	78±14
History of smoking	31,920	7,544 (33%)	1,507 (32%)	725 (18%)	94 (20%)
History of alcohol use	12,832	1,114 (15%)	241 (16%)	618 (15%)	
History of cocaine use	32,141	1,810 (8%)	404 (8%)	566 (14%)	
History of diabetes	35,528	496 (2%)	99 (2%)	144 (3%)	7 (2%)
History of hypertension	35,420	3,012 (12%)	598 (11%)	6,15 (15%)	33 (7%)
LVEF (%)	20,235	61·5±7·9	61·5±7·8	61·6±7·1	
Cytomegalovirus status	35,913	15,188 (60%)	3,138 (60%)	2,489 (62%)	778 (67%)
Creatinine (µmol/l)	31,558	105±84	106±82	115±98	
BUN (mg/dl)	31,262	14±11	15±12	17±14	
Serum bilirubin (mg/dL)	31,077	1·24±1·94	1·23±1·69	1·48±2·64	
Ischemic time (minutes)	36,276	186±65	186±65	197± 66	172± 66
Cause of donor death	47,785				
Head trauma		17,497 (51%)	3,673 (53%)	2,2330 (44%)	285 (22%)
Cerebrovascular event		10,482 (31%)	2,2064 (30%)	1,400 (26%)	733 (57%)
Other		6,227 (18%)	1,257 (18%)	1,570 (30%)	267 (21%)

Data are means (SD) or number (%).The numbers are calculated on patients with available data. BUN, blood urea nitrogen; LVEF, left ventricular ejection fraction; ISHLT, International Society for Heart and Lung Transplantation; IVC, internal validation cohort; NTTD, Nordic Thoracic Transplatation Database; TVC, temporal validation cohort.

S1D Table. Blood group, immunology and era of the study population from ISHLT registry and the Nordic Thoracic Transplantation Database.

		ISHLT			NTTD
	N	**Derivation cohort** N = 41,780	**IVC** N = 8,569	**TVC** N = 6,276	N = 1,285
Recipient blood group	57,910				
A		18,304 (44%)	3,752 (44%)	2,629 (42%)	618 (48%)
AB		2,737 (7%)	539 (6%)	500 (8%)	72 (6%)
B		5,300 (13%)	1,051 (12%)	859 (14%)	158 (12%)
O		15,439 (37%)	3,227 (38%)	2,288 (36%)	437 (34%)
Donor blood group	57,910				
A		16,802 (40%)	3,446 (40%)	2,315 (37%)	544 (42%)
AB		1,266 (3%)	257 (3%)	312 (5%)	20 (2%)
B		4,226 (10%)	806 (9%)	675 (11%)	132 (10%)
O		19,486 (47%)	4,060 (47%)	2,974 (47%)	589 (46%)
HLA-A mismatch	25,473				
0		735 (4%)	157 (4%)	111 (4%)	744 (%)
1		7,438 (41%)	1,514 (41%)	1,196 (40%)	339 (51%)
2		9,948 (55%)	2,044 (55%)	1,665 (56%)	282 (42%)
HLA-B mismatch	28,416				
0		285 (1%)	55 (1%)	51 (2%)	9 (1%)
1		5,304 (26%)	1,085 (26%)	821 (24%)	214 (34%)
2		1,4663 (72%)	3,010 (73%)	2,510 (74%)	409 (65%)
HLA-DR mismatch	25,385				
0		720 (4%)	163 (4%)	142 (5%)	19 (3%)
1		7,324 (41%)	1,496 (40%)	1,169 (40%)	339 (46%)
2		9,934 (55%)	2,060 (55%)	1,646 (56%)	373 (51%)
Era of transplantation	57,910				
1991-1995		6,029 (14%)	1,259 (15%)		
1996-2000		14,804 (35%)	2,949 (34%)		450 (35%)
2001-2005		13,078 (31%)	2,682 (31%)		451 (35%)
2006-2010		7,869 (19%)	1,679 (20%)	6,276 (100%)	384 (30%)

Data are means (SD) or number (%). The numbers are calculated on patients with available data. HLA, human leukocyte antigen; ISHLT, International Society for Heart and Lung Transplantation; IVC, internal validation cohort; NTTD, Nordic Thoracic Transplatation Database; TVC, temporal validation cohort.
