# Supplementary material for: The International Heart Transplant Survival Algorithm (IHTSA): A New Model to Improve Organ Sharing and Survival
Source: PLoS One. 2015 Mar 11;10(3):e0118644. doi: 10.1371/journal.pone.0118644 (PMC4356583; doi:10.1371/journal.pone.0118644)
Supplement: S2 Table — (PDF) [file pone.0118644.s003.pdf]

S2 Table. Time-dependent hazard ratios for different subgroups.

	N	Hazard ratio at 1 year	Hazard ratio at 5 years	Hazard ratio at 10 years
**Recipient**				
Age (per 8 years)				
18 – 30 years	2,796	0.95	0.96	1.02
30 – 45 years	6,992	0.99	1.02	1.10
45 – 60 years	21,053	1.06	1.11	1.20
> 60 years	10,939	1.12	1.19	1.24
Height (per 6 cm)				
< 160 cm	2,953	0.98	0.99	0.99
160 – 180 cm	26,664	0.99	0.99	1.00
> 180 cm	12,163	0.99	1.00	1.00
Weight (per 10 kg)				
< 70 kg	12,862	1.01	1.02	1.02
70 – 90 kg	19,829	1.02	1.02	1.02
> 90 kg	9,089	1.02	1.03	1.03
SPP (per 10 mmHg)				
< 45 mmHg	22,457	1.02	1.01	1.00
45 – 55 mmHg	9,161	1.02	1.01	1.00
> 55 mmHg	10,162	1.02	1.01	1.00
PVR (per wood units)				
< 2.5 wood units	25,016	1.01	1.00	1.00
2.5 – 5.0 wood units	13,450	1.01	1.00	1.00
> 5.0 wood units	3,314	1.01	1.00	1.00
Creatinine (per 22 µmol/l)				
< 100 µmol/l	18,060	1.03	1.02	1.01
100 – 150 µmol/l	17,288	1.03	1.02	1.01
150 – 200 µmol/l	4,115	1.03	1.02	1.01
> 200 µmol/l	2,317	1.02	1.01	1.00
Serum bilirubin (per 0.4 mg/dl)				
< 1.5 mg/dl	32,901	1.01	1.00	1.00
1.5 – 3 mg/dl	6,178	1.01	1.00	1.00
> 3 mg/dl	2,701	1.01	1.00	1.00
**Donor**				
Age (per 11 years)				
15 – 30 years	17,599	1.16	1.09	1.07
30 – 50 years	18,564	1.19	1.12	1.08
50 – 70 years	5,617	1.21	1.14	1.09
Weight (per 10 kg)				
< 70 kg	12,381	0.99	1.01	1.03
70 – 90 kg	20,820	0.99	1.02	1.03
> 110 kg	8,579	1.00	1.02	1.03
Duration of ischemia (per 42 min)				
< 2 h	6,335	1.02	1.00	1.00
2 – 4 h	27,586	1.03	1.01	1.00
> 4 h	7,859	1.03	1.01	1.00

The data are hazard ratio for the different time points. CODD, cause of donor death; ITHSA, international heart transplantation survival algorithm.
