# Supplementary material for: The International Heart Transplant Survival Algorithm (IHTSA): A New Model to Improve Organ Sharing and Survival
Source: PLoS One. 2015 Mar 11;10(3):e0118644. doi: 10.1371/journal.pone.0118644 (PMC4356583; doi:10.1371/journal.pone.0118644)
Supplement: S3 Table — Influences of the allocated organs, and the predicted and observed survival time depending on the waiting list size, using the IHTSA model, Clinical model respectively by random. A) Applied on the internal validation cohort. B) Applied on the external validation cohort, Nordic Thoracic Transplantation Database. (PDF) [file pone.0118644.s004.pdf]

S3A Table. Influences of the allocated organs, and the predicted and observed survival time depending on the waiting list size, using the IHTSA model, Clinical model respectively by random tested on the internal validation cohort (IVC).

Allocated organs N (%)				Predicted median survival time (months)			Observed median survival time (months)		
N	IHTSA	Clinical	p Value†	IHTSA	Clinical	p Value‡	IHTSA	Clinical	p Value§
10	3598 (84%)	2842 (65%)	<0·001	164	158	<0·001	145	136	0·089
30	3913 (93%)	3555 (82%)	<0·001	164	157	<0·001	148	134	0·023
50	4123 (94%)	3623 (86%)	<0·001	164	157	<0·001	145	142	0·104
100	4103 (97%)	3861 (91%)	<0·001	165	156	<0·001	148	133	0·004
200	4281 (98%)	4107 (95%)	<0·001	166	157	<0·001	151	143	0·030
N	IHTSA	Random	p Value†	IHTSA	Random	p Value‡	IHTSA	Random	p Value§
10	3598 (84%)	3661 (85%)	0·317	164	136	<0·001	145	128	<0·001
30	3913 (93%)	4008 (92%)	0·591	164	133	<0·001	148	122	<0·001
50	4123 (94%)	3929 (94%)	0·535	164	129	<0·001	145	122	<0·001
100	4103 (97%)	4178 (97%)	0·790	165	126	<0·001	148	122	<0·001
200	4281 (98%)	4102 (98%)	0·886	166	127	<0·001	151	118	<0·001

Data are number (%) or median survival time. †χ^2 test, ‡Mann-Whitney test and §log-rank test.

S3B Table. Influences of the allocated organs, and the predicted and observed survival time depending on waiting list size, using the IHTSA model, Clinical model respectively by random applied on the external validation cohort (Nordic Thoracic transplantation Database).

Allocated organs N (%)				Predicted median survival time (months)			Observed median survival time (months)		
N	IHTSA	Clinical	p Value†	IHTSA	Clinical	p Value‡	IHTSA	Clinical	p Value§
10	571 (88%)	427 (66%)	<0·001	167	165	0·020	160	135	0·679
30	590 (95%)	535 (82%)	<0·001	169	163	<0·001	159	144	0·182
50	622 (97%)	531 (85%)	<0·001	173	165	<0·001	159	149	0·671
100	595 (97%)	598 (93%)	0·001	172	163	<0·001	159	157	0·562
200	619 (96%)	620 (93%)	0·013	174	160	<0·001	160	149	0·381
N	IHTSA	Random	p Value†	IHTSA	Random	p Value‡	IHTSA	Random	p Value§
10	571 (88%)	574 (90%)	0·277	167	142	<0·001	160	141	0·091
30	590 (95%)	631 (96%)	0·476	169	140	<0·001	159	144	0·100
50	622 (97%)	627 (97%)	0·760	173	139	<0·001	159	141	0·016
100	595 (97%)	650 (97%)	0·971	172	136	<0·001	159	138	0·011
200	619 (96%)	611 (95%)	0·160	174	139	<0·001	160	143	0·063

Data are number (%) or median survival time. †χ^2 test, ‡Mann-Whitney test and §log-rank test.
